# A Sequential Electrospinning of a Coaxial and Blending Process for Creating Double-Layer Hybrid Films to Sense Glucose

**DOI:** 10.3390/s23073685

**Published:** 2023-04-02

**Authors:** Yutong Du, Zili Yang, Shixiong Kang, Deng-Guang Yu, Xiren Chen, Jun Shao

**Affiliations:** 1 School of Materials and Chemistry, University of Shanghai for Science and Technology, Shanghai 200093, China; 213353175@st.usst.edu.cn (Y.D.); 203613094@st.usst.edu.cn (Z.Y.);; 2 Shanghai Engineering Technology Research Center for High-Performance Medical Device Materials, Shanghai 200093, China; 3 Shanghai Institute of Technical Physics, Chinese Academy of Sciences, 500 Yutian Road, Shanghai 200083, China

**Keywords:** coaxial electrospinning, core–sheath nanofibers, glucose sensor, colorimetric sensing, structural hybrids

## Abstract

This study presents a glucose biosensor based on electrospun core–sheath nanofibers. Two types of film were fabricated using different electrospinning procedures. Film F1 was composed solely of core–sheath nanofibers fabricated using a modified coaxial electrospinning process. Film F2 was a double-layer hybrid film fabricated through a sequential electrospinning and blending process. The bottom layer of F2 comprised core–sheath nanofibers fabricated using a modified process, in which pure polymethacrylate type A (Eudragit L100) was used as the core section and water-soluble lignin (WSL) and phenol were loaded as the sheath section. The top layer of F2 contained glucose oxidase (GOx) and gold nanoparticles, which were distributed throughout the polyvinylpyrrolidone K90 (PVP K90) nanofibers through a single-fluid blending electrospinning process. The study investigated the sequential electrospinning process in detail. The experimental results demonstrated that the F2 hybrid film had a higher degradation efficiency of β-D-glucose than F1, reaching a maximum of over 70% after 12 h within the concentration range of 10–40 mmol/L. The hybrid film F2 is used for colorimetric sensing of β-D-glucose in the range of 1–15 mmol/L. The solution exhibited a color that deepened gradually with an increase in β-D-glucose concentration. Electrospinning is flexible in creating structures for bio-cascade reactions, and the double-layer hybrid film can provide a simple template for developing other sensing nanomaterials.

## 1. Introduction

Today, diabetes has become a great concern in the medical community. The treatment process of diabetic patients often involves monitoring their urinary sugar and blood glucose levels. The latest developed glucose uptake probes can be used in both in vivo and in vitro bioluminescent detection. They are new tools to identify glucose transport inhibitors with fine detection accuracy, and can be widely used in the field of drug development as the imaging reagent [1]. In complex cases, glucose sometimes needs to be detected in different parts. The glucose detection technology in biofluidics is relatively mature [2]. Recently, glucose detection has been successfully achieved in the mouse brain [3]. Materials used in glucose sensors involve metals [4], polymers [5], and composites [6]. Nanomaterials have played a pivotal role in biosensing technology development [7]. Nanostructures are advancing biosensing technology by breaking electron transport barriers and redefining critical points of structural properties and sensing accuracy [8]. Electrochemical sensing technology is widely used in various fields, with glucose sensing being the most common. However, the use of electrochemical measurement techniques is limited because of the requirement for various instruments [9]. Improving utilization often requires other ways to participate in the biosensing system without deteriorating sensing performance.

Electrospinning technology simplifies the preparation of nanofibers, expands its applications, and enables biomolecular detection without complex instruments [10,11,12,13]. Electrospun devices can be created from many substances, such as polymers, small molecules, colloidal particles, and complexes [14,15,16,17,18]. Filament formation can be enabled by increasing the viscoelasticity of the liquid through physical or chemical modification [19,20,21]. Single-fluid electrospinning is the most common and low-cost method, involving only one spinnable fluid with its molecular weight determining the electrospinnability [22,23]. However, single-fluid electrospinning produces monoaxial nanofibers with a uniform structure, limiting their ability to generate multilevel effects or regulate the spatial relationship between components, let alone design multiple functions, as compared with multiple-fluid electrospinning [24,25,26,27,28]. A multilevel structure enables effective design and regulation of components’ places and distributions, achieving customized functionality at the nanoscale [29,30]. Multifluid spinning improves the efficiency of preparing functional nanofibers, allowing for one-step preparation of complex functional nanofibers [31,32,33,34,35,36,37,38,39]. A series of spinnerets with multistage structures, which could be used to implement multi-fluid electrospinning processes for preparing complex biomedical nanostructures, were reported [40,41,42,43,44,45]. Core–sheath nanofibers are nanomaterials with a hierarchical structure that can be customized for specific functions [46]. The coaxial electrospinning process increases material selection and involves non-spun fluids in the electrospinning process, further enhancing the potential for functional fiber preparation [47,48,49]. Electrospun fibers can be improved through post-treatments, such as chemical and physical treatments, including carbonization, heat treatment, and crosslinking. Crosslinking is particularly crucial for enhancing the mechanical and electrical properties of disordered monoaxial nanofibers, while also maximizing their morphology and complexity. Similarly, the heating temperature or saturated steam method can be used to improve the properties of polymer fibers [50,51]. Electrospun nanofibers have been demonstrated to be useful in biomedical field, such as biosensing [52,53], clinical diagnosis and treatment [54,55], and drug delivery. They can be tailored to perfectly adapt to various complex biological environments, making them a valuable tool in the field of biomedical engineering.

The sensors used for glucose detection include enzymatic and non-enzymatic ones. Enzymatic sensors typically use glucose oxidase, glucose dehydrogenase, and hexokinase, while non-enzymatic sensors use various electrocatalysts [56]. Enzyme-based glucose sensors have been extensively researched since the 1960s and remain a popular focus of study today [57]. New materials and electrospinning techniques have increased the efficiency of glucose detection using bioenzymes. For instance, Manesh et al. obtained a glucose electrode by fixing GOx into a composite fiber membrane composed of polymethyl methacrylate (PMMA), multi-wall carbon nanotubes (MWCNT), and poly(diallyl dimethylammonium chloride) (PDDA) [58]. The glucose electrode showed excellent electrocatalytic activity against hydrogen peroxide. Metal oxide electrospun fibers can achieve similar functions and faster response speeds [59]. Non-enzymatic sensors have also been studied because of issues with enzyme activity being affected by external factors [60]. Mian et al. fixed and embedded a series of bimetal nanoparticles MCo (M = Cu, Fe, Ni, and Mn) into carbon nanofibers (CFs) because of the structural advantage of 3D network films and the synergistic effect of Co(III)/Co(IV) and Cu(II)/Cu(III) redox electric couples [61]. Enzyme immobilization techniques aim to maintain enzyme stability for effective biocatalytic activity. Techniques include covalent binding [62], adsorption [63,64], crosslinking [65], and encapsulation [66]. Crosslinking with glutaraldehyde (GA) and quinapril can effectively increase enzyme stability and recycling [67]. Encapsulation is the most widely used method but has limitations such as enzyme leakage and low material transport efficiency. Electrospun fibers can efficiently load various biological enzymes and significantly improve catalytic efficiency [68,69]. However, enzymes are sensitive and prone to self-dissolution or destruction due to the large number of residues on their surface and sensitive active center. Thus, they must be dissolved in water for electrospinning and loaded onto a water-soluble polymer matrix, such as polyvinylpyrrolidone (PVP) or polyvinyl alcohol (PVA), either through direct spinning, physical adsorption, or covalent action. Water-soluble lignin (WSL) is derived from plant materials, making it non-toxic and unlikely to elicit an immune response in living organisms. Plant materials are also renewable resources, making WSL a sustainable option. WSL-based sensors have been shown to exhibit high sensitivity [59], capable of detecting very small amounts of target molecules or ions. Enzymes loaded in fibers must be immobilized to maintain stability, and GA crosslinked nanofibers offer better hydrophobicity. Using nanofibers as nanoreaction vessels can enhance enzyme reaction kinetics, reactivity, and selectivity. However, electrospinning may irreversibly change the enzyme’s configuration, leading to poor catalytic performance, which can be avoided by reducing the electric field strength.

In this study, a new electrospinning strategy was developed for implementing simple and efficient glucose sensing. A modified coaxial process and a single-fluid process were combined to tailor the spatial position and distribution of components in an electrospun bi-layer fibrous film, which showed a better functional performance than a sole core–sheath fibrous mat. Poly(methyl methacrylate) A-type (Eudragit L 100) and polyvinylpyrrolidone K90 (PVP K90) were selected as the polymeric matrices owing to their filament-forming properties. Eudragit L 100 can immobilize enzymes on the nanofibers’ surfaces, protecting them from degradation while allowing for selective interaction with the target analyte. WSL can enhance enzyme activity and protect the enzyme from denaturation. PVP is biocompatible and water-soluble, making it an ideal candidate for use in enzyme immobilization and stabilization. When combined with enzymes, PVP can help protect them from denaturation, maintain their activity, increase their shelf life, and improve their performance. Glucose oxidase (GOx) and phenol are used as the functional ingredients for the first and second levels of the biological reaction to construct a colorimetric glucose sensor. The bi-layer sensors were systematically characterized and were verified to be able to provide a basis for accurate reactions.

## 2. Materials and Methods

### 2.1. Materials

Polymethylacrylate (Eudragit L100, EL100) was purchased from Shanghai Chineway Pharmaceutical Technology Co., Ltd., Shanghai, China. Polyvinylpyrrolilione K90 (PVP K90) and glucose oxidase (GOx, 50KU) were purchased from Sigma Aldrich (Shanghai) Trading Co., Ltd., Shanghai, China. Phenol, ethanol, and glutaraldehyde (GA, 25 wt%) were purchased from Sinopharm Chemical Reagent Co., Ltd., Shanghai, China. β-D-Glucose, citric acid (C_6_H_8_O_7_•H_2_O), and sodium hydroxide (NaOH) were purchased from Shanghai Aladdin Biochemical Technology Co., Ltd. Water-soluble lignin (WSL) and gold nanoparticle (DAMP) were provided by the School of Chemical Engineering, Nanjing Forestry University. More details about WSL can be found in Ref. [59].

### 2.2. Fabrication of the Core–Sheath and Hybrid Films

A customized detachable concentric spinneret was explored to prepare all the samples. Aluminum foil was used as a fiber collector. The preparation parameters of the only core–sheath fiber films F1 and double-layer hybrid films F2 (composed of a bottom layer of core–sheath nanofibers and a top layer of monoaxial nanofibers) are shown in Table 1. The environmental conditions included a temperature of 25 ± 3 °C, a relative humidity of 50 ± 5%, and no strong magnetic field and large wind direction interference around the spinning experiment device. A volume of 20 mL 25% (*w*/*v*) GA aqueous solution was diluted to 2%, and the steam produced by the aqueous solution was used to cross-link the two trimmed fiber membranes for 10 min. Thereafter, the membranes were dried at room temperature.

### 2.3. Characterization of Nanofiber Membrane Properties

#### 2.3.1. Morphology and Physical State

The surface morphology of nanofibers was observed by field-emission scanning electron microscopy (FESEM, Quanta FEG450, Hillsboro, OR, USA) and transmission electron microscopy (TEM, JEM 2100 F, Tokyo, Japan). The fiber was coated with sputtered gold for 120 s under a nitrogen atmosphere, and then the surface morphology of the fiber was observed under an accelerating voltage of 20 kV. The diameter distribution of the fibers was analyzed using the ImageJ software. The microregion composition of the fiber membrane surface was analyzed using an energy-dispersive spectrometer (EDS, Quanta FEG450, USA) and fluorescence microscopy (FM, M205FA, Germany) was used to perform UV-C irradiation (405 nm wavelength) imaging of the WSL component in the fiber membrane. The physical states of the raw materials and nanofiber membranes were investigated using X-ray diffraction (XRD) patterns, using an AXS X-ray diffractometer (XRD; Bruker, Bremen, Germany) from a diffraction angle (2θ) of 10° to 60° with Cu Ka (l = 1.541 Å) radiation. Fourier transform infrared spectroscopy (FTIR, SPECTRUM 100, USA), substance long-wave absorption peaks of the raw materials, and nanofiber membranes were detected by using KBr as a reference material for transmission scanning. The scanning range was set to 450–4000 cm^−1^ with a resolution of 2 cm^−1^.

#### 2.3.2. Glucose Degradation Test

Citric acid (21 g) was dissolved in 75 mL of boiling distilled water, and 7.45 g of NaOH was added. The mixture was then transferred to a volumetric flask and filled up to 100 mL with distilled water, resulting in a buffer solution with a pH of 4.45. A buffer system with a pH of 5.0 was prepared by multiple dilutions and verified using a pH meter (PH-100). Glucose solutions with concentrations of 10, 20, 30, and 40 mmol/L were prepared at pH 5.0 and the temperature was set to 50 °C with an agitation rate of 50 rpm. The composite fiber membrane was placed in the prepared solution.

### 2.4. Glucose-Sensing Performance Detection

The glucose solution system with a pH of 5.0 and concentrations of 1, 5, 15, and 20 mmol/L was prepared. The composite fiber film was cut into pieces of 9 cm^2^ and placed into the prepared solutions. After the color development reaction (3 h), the color of the solution was recorded using a camera. The RGB color was then converted to HSL, and linear fit was performed on the color phase angles.

## 3. Results and Discussion

### 3.1. Implementation of Electrospinning

**Figure 1:**
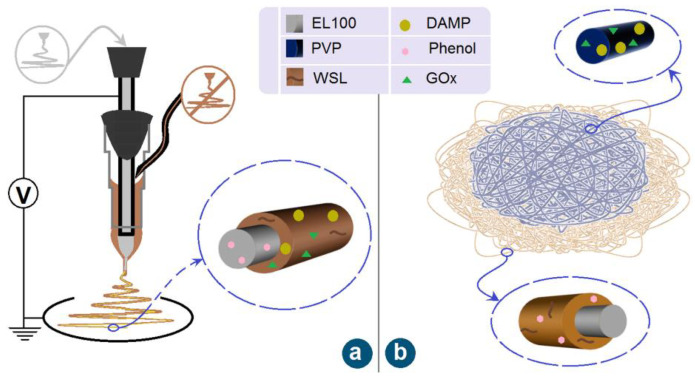
The modified coaxial electrospinning process (**a**) and the sequential collections of core–sheath and monolithic nanofibers (**b**).

Electrospinning is popular for creating nanofibers of polymers. Later, attention shifted to fabricating complex structures and treating unspinnable fluids [70,71,72]. Deposited nanofibers can also be organized to prepare functional fiber films [73]. Shown in Figure 1a is a diagram of a modified coaxial electrospinning process, by which unspinnable WSL, DAMP, and GOx can be treated to a sheath section on the core nanofibers containing EL100 and phenol. The films F1 were thus directly fabricated.

Electrospinning is facile for tailoring components and compositions on a nano scale through both working fluid preparations and fiber collection. Figure 1b is a diagram of the preparation strategy. A double-layered hybrid film F2 can be prepared using a sequential electrospinning combining a typical modified coaxial process and a later single-fluid blending process. The GOx and DAMP can be separated from the electrospun core–sheath nanostructure. 

**Figure 2:**
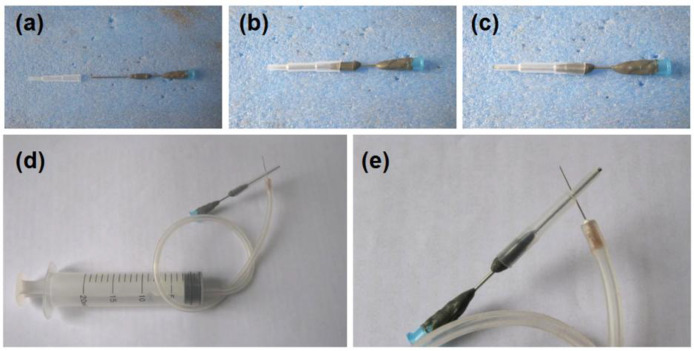
The preparation of the detachable concentric spinneret: (**a**–**c**) shows its formation by connecting a PE tubing with a traditional needle; (**d**,**e**) show the organization of syringes for conducting the working processes.

In an electrospinning apparatus, the spinneret is the most important element and is full of innovations [74]. In literature, almost all the concentric spinnerets are prepared using metals (mostly stainless steel) because of the fine conductivity. In this study, a special concentric spinneret was developed, in which a PE tube was explored as the sheath section. The preparation procedure was shown in Figure 2. A section of the metal capillary was covered by epoxy resin for fixing the PE tubing, as indicted from Figure 2a–c. Later, a syringe could be directly inserted in the spinneret for pumping the core working fluid. Another syringe could be connected with the spinneret for driving the sheath fluid through a high elastic silicon tubing and a sharp needle (Figure 2d,e).

**Figure 3:**
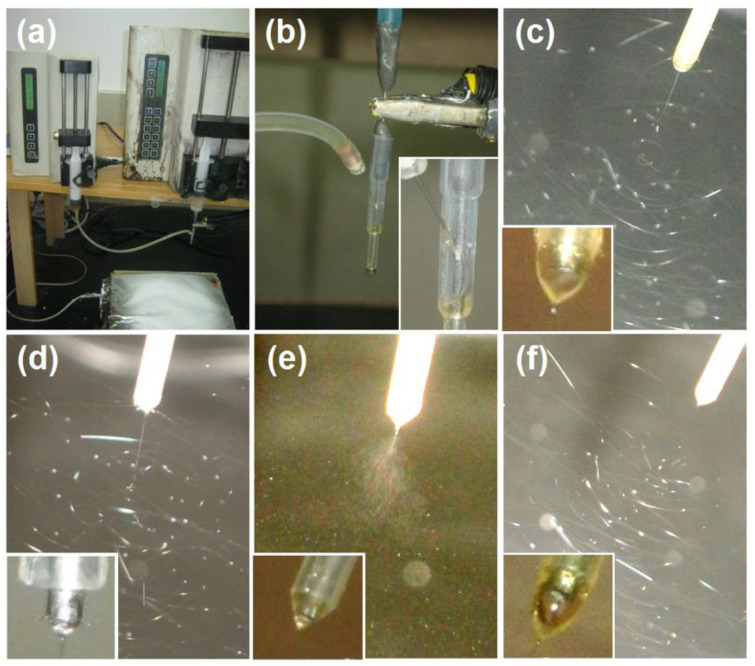
Implementations of EHDA processes for fabricating samples F1 and F2: (**a**) a whole digital image of the customized electrospinning apparatus; (**b**) the connection of spinneret with the working fluid (the down-right inset shows an enlarged image) and power supply; (**c**) the preparation of core–sheath nanofiber F1 (the down-left inset shows a typical Taylor cone); (**d**) a single-fluid electrospinning of the core working fluid, which had fine electrospinnability (the down-left inset shows a typical Taylor cone); (**e**) a single-fluid electrospraying of the sheath working fluid, which was not electrospinnable (the down-left inset shows a typical Taylor cone); and (**f**) the preparation of the down layer of sample 2 (the down-left inset shows a typical compound Taylor cone).

The working processes for preparing films F1 and F2 are recorded in Figure 3. Shown in Figure 3a is a digital photo of the electrospinning apparatus and the working process for creating only core–sheath fiber film F1. Figure 3b is a picture showing the connection of spinneret with the working fluid and power supply through an alligator clip. The down-right inset shows an enlarged image of the pumping process of sheath working fluid. A typical process of the coaxial electrospinning for preparing films was recorded and shown in Figure 3c, in which three steps, i.e., a compound Taylor cone (the bottom-left inset), a straight fluid jet, and a bending and whipping instable region, are obvious. When the sheath fluid was stopped, the coaxial process was transferred into a single-fluid blending process (Figure 3d) with a small Taylor cone from the core metal capillary nozzle (the bottom-left inset of Figure 3d). This indicates that the core fluid had a fine electrospinnability. However, when the core fluid was stopped, the coaxial process was converted to a typical electrospraying process (Figure 3e; the bottom-left inset shows the Taylor cone). For the preparation of film F2, the coaxial process in creating the bottom layer is exhibited in Figure 3f; a typical three-stage process and a clear compound Taylor cone (the bottom-left inset) are clear.

### 3.2. Morphology and Structure of Nanofibers and the Components’ State and Compatibility

**Figure 4:**
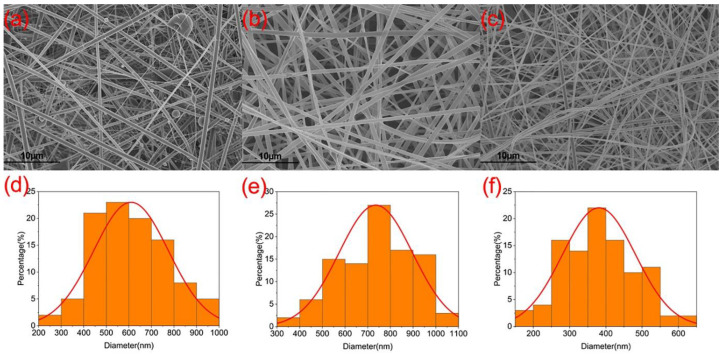
The SEM images of the fiber membranes and the corresponding fiber diameter distribution: (**a**) core–sheath fiber membrane F1; (**b**) bottom layer of hybrid membrane F2; (**c**) top layer of hybrid membrane F2; (**d**) diameter distribution of core–sheath nanofiber F1; (**e**) bottom-layer fiber diameter distribution of hybrid film F2; (**f**) top-layer fiber diameter distribution of hybrid film F2.

Electrospinning is a versatile technique that can be used to fabricate functional nanomaterials through the encapsulation of functional ingredients [75,76,77,78,79]. The morphology of the resulting nanofibers is largely determined by the filament-forming polymeric matrix [80,81]. Here, the host polymers PVP and EL100 have fine electrospinnability. The microscopic configuration and morphology of hybrid nanofiber membrane F2 can be revealed by SEM and fluorescence microscopy, and the functional support of the membrane layer can be structural. The SEM images of the only core–sheath structure F1, the bottom layer and the upper layer of hybrid fiber membrane F2, and their corresponding diameter and size distribution are included in Figure 4. As can be seen from the figures, the morphology of the fibers in the only core–sheath structure varied greatly from the fibers in hybrid membrane fiber F2. Hybrid membrane fibers F2 had a smooth surface, with an average diameter of 500–1000 nm for the bottom-layer fibers and 250–550 nm for the top-layer fibers. The fibers of only a core–sheath structure carried some aggregated particles, and the presence of these particles indicates that the addition of material in the sheath affects the continuity of the spinning process, resulting in partially beaded fibers and irregular particles deposited on the fibers. The average fiber diameter of the only core–sheath structure was 400–800 nm, which is smaller overall compared with the fiber diameter of the coaxial part of the bottom layer of hybrid membrane F2, indicating that the amount of functional material added in the sheath of the modified coaxial electrospun fiber layer may be negatively correlated with the final fiber diameter size.

**Figure 5:**
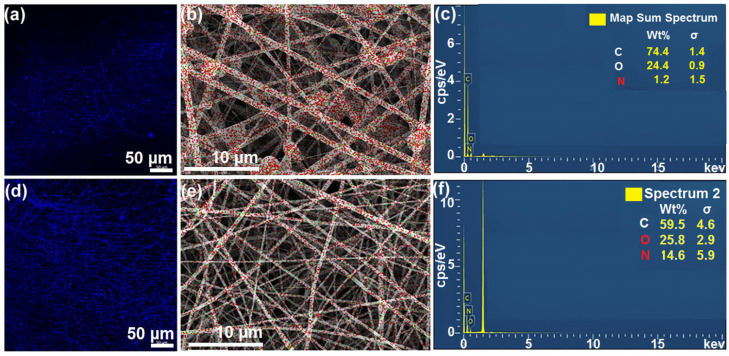
Fiber membrane fluorescence photo and EDS energy spectrum: (**a**) WSL fluorescence photo in core–sheath structure fiber membrane F1; (**b**,**c**) N element energy spectrum in core–sheath fiber membrane F1; (**d**) WSL fluorescence photo in core–sheath layer of hybrid fiber membrane F2; (**e**,**f**) N element energy spectrum in the whole hybrid fiber membrane F2.

In order to verify whether the modified coaxial electrospinning technology can successfully convert the non-spinning fluid into solid fibers, fluorescence microscopy can be used to observe the images of WSL in the two kinds of nanofibers, and EDS can show the N element distribution of the fiber membrane. Figure 5 presents the WSL fluorescence photo and N element distribution of the two fiber membranes. From Figure 5a,b, it can be observed that the WSL in fiber distribution was more uniform and continuous in the bottom layer of hybrid film F2, which is consistent with the previous analysis. The fluid could not be completely transformed into filaments and lost a certain amount, and the remaining liquid solidified into granular particles that scattered between the fibers. However, because of the low resolution in the fluorescence photos, the image could not appear. Figure 5b–f show the N element distribution of fiber membranes. The N element energy spectrum indicates that both fiber membranes contained GOx, which has been reported to be spun into nanofibers in a previous study [82]. However, because the structure and polymer substrate of GOx were different in the two fiber membranes, the content of GOx in the different fibers was different. On one hand, in an only core–sheath structure, dissolved GOx and WSL, and WSL, because of its low molecular weight, could not bind a large number of GOx molecules together, resulting in GOx being lost as droplets during coaxial electrospinning. This was not the case in the hybrid membrane, where PVP K90 could embed GOx molecules between its chains and maximize the preservation of GOx content. On the other hand, PVP K90 in the hybrid membrane layer contained a certain amount of N elements, so the total amount of N elements in the two parts was greater than the N content in the only core–sheath structure F1.

**Figure 6:**
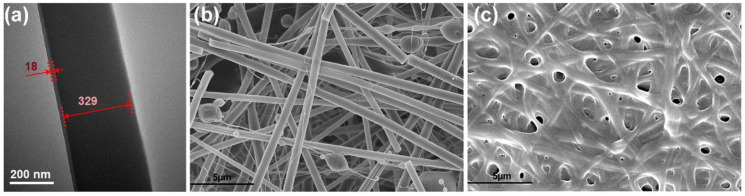
(**a**) The TEM micrographs of the core–sheath fiber membrane; the SEM micrographs of the fiber membrane after crosslinking: (**b**) only core–sheath fiber membrane F1; (**c**) hybrid fiber membrane F2.

As shown in Figure 6a, the structure of the core–sheath fiber was observed using TEM. The fiber membrane prepared by coaxial electrospinning technology had a core–sheath structure, and the diameter of the core layer of the fiber in the figure was about 329 nm, while the sheath layer thickness was about 18 nm. This fiber belongs to a core–sheath structured nanofiber with a relatively thin sheath layer. The crosslinking of fiber membranes can greatly increase the dissolution time of polymers and improve the connection between GOx, promoting electron transfer and active site exposure. This is especially important for hydrophilic polymers such as PVP, which dissolve quickly in water. If PVP dissolves, GOx becomes free in the solution, making it difficult to achieve the maximum catalytic effect. Figure 6 illustrates crosslinked SEM micrographs of the two fiber membranes. From Figure 6b, it can be observed that the fiber broke after 10 min of GA steam because of the thin shell layer and the presence of hydrophobic polymer EL100 in the fiber body. Moreover, the amount of GOx in the shell layer was weakened at room temperature. In contrast, the hybrid fiber membrane with PVP layer underwent significant morphological changes after GA steam crosslinking. The fibers were connected, and some fibers partially dissolved and collapsed (Figure 6c). This is mainly because PVP absorbs water from the GA steam, causing the PVP molecules to unwind and increase the fiber volume. As a result, GOx molecules were released from the chain, and the amino group reacted with GA to connect the GOx. However, excessive protein degeneration occurred, so the time was controlled within 10 min.

The WSL was taken from the prehydrolysate of the wood, where the dissolved sugars and the WSL are difficult to separate and recover simultaneously [83]. The WSL was isolated from the prehydrolysate by using an adsorbent and hydrophobic XAD resin (poly(styrene-co-divinylbenzene)), and the composition of the resulting WSL is shown in Table 2. It can be seen that the WSL contained a high content of the lignin fraction (84.5%). However, 7.7% of the carbohydrates (5.6% xylan and 2.1% glucan) remained in the purified WSL. These carbohydrates are thought to be due to covalent junctions in lignin–carbohydrate (LC). Related studies also found that the reflux fluid from the pre-hydrolysate flowing through the XAD resin represents the fraction of the enriched lignin–carbohydrate complex, which was identified by using the LC bond signal evident in the 2D-NMR spectra [84]. It should be noted that the particle size of WSL was smaller than the lignin nanoparticles (80–200 nm) [85] because WSL is the product of lignin prehydrolysate at high temperatures (above 170 °C).

**Figure 7:**
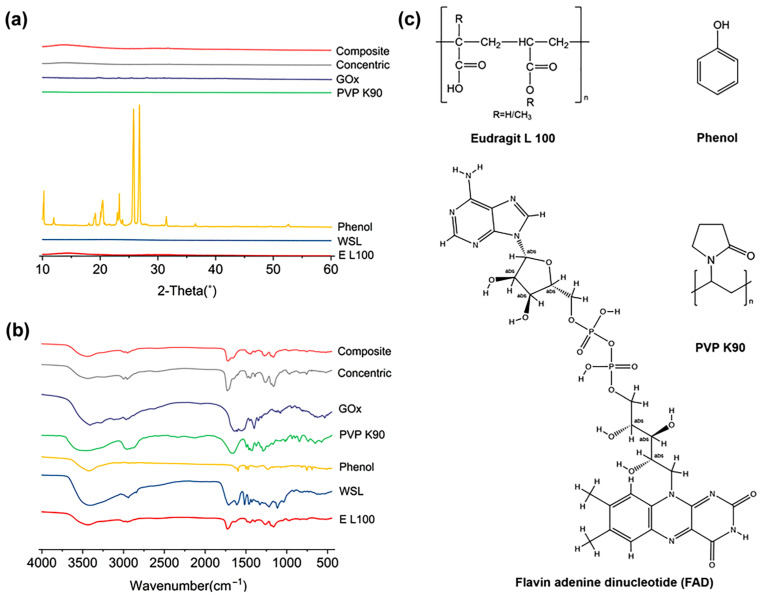
Physical state and compatibility characterization: (**a**) XRD pattern; (**b**) FTIR spectra; and (**c**) chemical structure formula of the raw materials.

XRD analysis and infrared band scanning can determine whether the state of each component underwent a significant transition and the possible connections between the main functional groups. Figure 7 shows the XRD and FTIR spectra of the raw materials and fiber membranes. As shown in Figure 7a, only phenol was crystalline in the raw materials, while the others were amorphous substances. There were no sharp Bragg diffraction peaks in the XRD patterns of the two fiber membranes, indicating that all substances existed in the fibers in an amorphous form. According to Figure 7b, EL 100 had an O-H vibration in the range of 3750–3000 cm^−1^, C-H in 3000–2700 cm^−1^, C=O in 1755–1670 cm^−1^, 1470–1000 cm^−1^ was the fingerprint area, and C-H vibration occurred in C-H and C-C single-bond backbone vibration. WSL also had O-H expansion vibration in the range of 3750–3000 cm^−1^, but it was relatively strong because of its diverse and complex substructures. CH expansion vibrations in the range of 3000–2700 cm^−1^ are mainly C-H-CH_2_ expansion vibrations at 2941 cm^−1^ and 2844 cm^−1^. The C=O expansion vibration in the range of 1755–1670 cm^−1^ was strong, mainly for the aromatic aldehydes at 1711 cm^−1^. The double-bond telescopic vibration in the range of 1690–1500 cm^−1^ was mainly that of the benzene ring skeleton at 1612 cm^−1^ and 1517 cm^−1^. In the range of 1470–1000 cm^−1^, there were mainly alkyl groups at 1463 cm^−1^ and aromatic ether at 1279 cm^−1^. Phenol had O-H expansion vibration at 3426 cm^−1^, benzene ring skeleton vibration in the range of 1620–1450 cm^−1^, and C-O expansion vibration at 1227 cm^−1^. PVP K90 had N-H expansion vibration in the range of 3750–3000 cm^−1^, CH_3_ and CH_2_- in the range of 3000–2700 cm^−1^, C=O in the range of 1755–1670 cm^−1^, and C-C single-bond backbone vibration in the range of 1470–1000 cm^−1^. GOx had N-H expansion vibration at 3410 cm^−1^, C-H expansion vibration at 3119–2970 cm^−1^, C=N expansion vibration at 1638 cm^−1^, benzene ring skeleton vibration at 1608 cm^−1^, and CH_3_ expansion vibration at 1400 cm^−1^. Comparing the raw materials with the fiber membranes, the absorption peaks of O-H and N-H in the range of 3750–3000 cm^−1^ were shifted and relatively weakened in both fiber membranes. The C=O absorption peak in the range of 1755–1670 cm^−1^ was also shifted, and the fingerprint region became shorter. The absorption peaks at 3439 cm^−1^ of the two membranes showed that the association of hydrogen bonding between multiple molecules occurred within the membrane. The absorption peak strength of the single core–sheath structure was higher than that of the composite membrane layer, which is because of the large variety of sheath materials and the number of hydrogen bonds formed between different molecular structures and functional substances of WSL. Therefore, it can be concluded that there is no unstable secondary reaction between the components in the fiber membrane, and a certain amount of associated hydrogen bonds would be produced between different molecules, ensuring the stability and good compatibility of the components in the fiber.

### 3.3. Glucose Degradation Test

**Figure 8:**
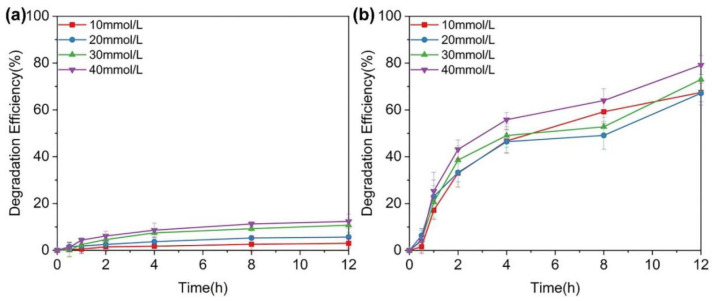
The degradation of β-D-glucose (n = 3): (**a**) single core–sheath fiber membrane; (**b**) composite fiber membrane.

GOx-containing fiber membranes in glucose solution can catalyze the degradation of glucose molecules by reacting with O_2_. Figure 8 shows the efficiency diagram of the degradation of β-D-glucose by two different fiber membranes. According to Figure 8a, the fiber membrane with a single core–sheath structure was extremely slow in degrading four different concentrations of β-D-glucose within 12 h. However, the overall trend for glucose degradation by the fiber membrane indicated that higher substrate concentrations are correlated with greater amounts of degradation. Based on the previous analysis results, the loaded GOx in the shell layer may be small and impossible to crosslink, leading to the fiber sheath dissolving quickly after contact with water. As a result, the GOx forms a free state in solution, leading to an overall insufficient activity and low degradation efficiency. From Figure 8b, the composite fiber membrane degraded the four concentrations of glucose molecules much more efficiently than the single core–sheath structure. The greater the glucose concentration is, the higher the final degradation efficiency is. This is because of the higher substrate concentration forming more effective binding with GOx and enhancing the stability of GOx. At β-D-glucose concentrations greater than 10 mmol/L, the amount of glucose degraded in the solution increased in a step fashion at different degradation times. The solution efficiency of 10 mmol/L was lower than the other three concentrations in the first hour, and after 2 and 4 h, it reached 30 and 45% for 10 mmol/L glucose, respectively, similar to that of 20 mmol/L, and exceeded the degradation efficiency of 20 and 30 mmol/L at 8 h. Finally, it reached more than 60% at 12 h, similar to the solution system efficiency with the concentration of 20 mmol/L. At the same time, it can be seen that the degradation efficiency of glucose molecules by the fibrous membrane was the lowest at 0.5 h. This is because effective binding between the substrate and the enzyme crosslinked in the fiber requires a certain amount of time. Only after that can the active site of GOx be activated, and then the catalytic reaction can begin completely.

### 3.4. Glucose-Sensing Performance Detection

**Figure 9:**
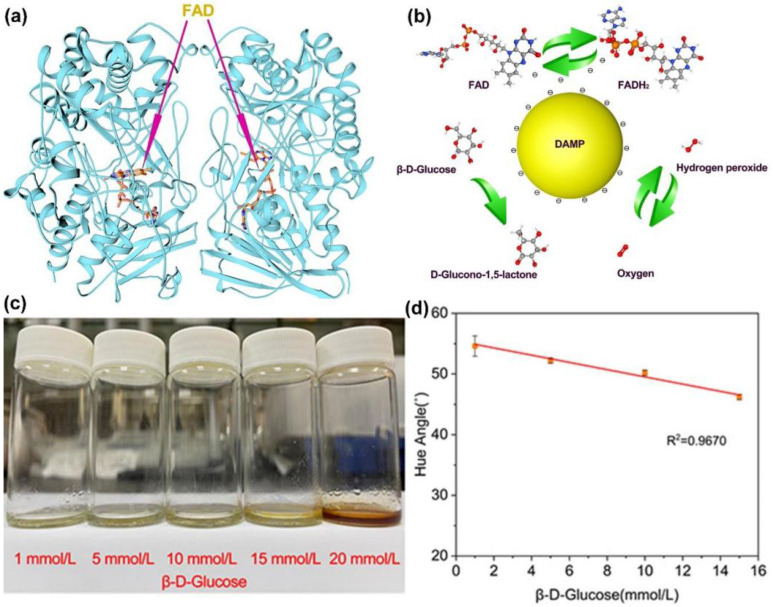
(**a**) Ribbon structure diagram of GOx homodimer; (**b**) diagram of the catalytic mechanism of DAMP; (**c**) the colorimetric sensing photo of glucose; (**d**) the linear fit for color phase angles in the range of 1–15 mmol/L (n = 5).

After using two types of fiber membranes for the catalytic degradation of glucose molecules, the composite membrane layer was selected for color sensing at low concentrations. This was because of the vapor crosslinking of the upper composite membrane layer, which caused a half immobilization effect on GOx. Although the fixed fiber membrane would still dissolve in an aqueous solution, GA simultaneously connected GOx and PVP, resulting in a longer dissolution time than before, which extended the GOx catalytic reaction time of efficient glucose molecules. The first-order biological reaction was protected, and the activity of GOx was well preserved. As GOx is a biologically active substance, its structure has a great influence on the active center. Figure 9a illustrates the ribbon structure diagram of the GOx homodimer. GOx is a protein, and the diagram, excluding FAD, is a polysome formed by 20 different amino acids, which is also the “coat” of FAD. FAD is the active center of GOx and is a functional molecule that can catalyze a material’s oxidation reaction. It is sensitive to temperature and pH environments. In the absence of substrate, GOx (FAD)’s suitable temperature is 30–60 °C, the pH range is 3.5–6.5, and the most suitable pH is 5.0. GOx; in 6 nm × 5.2 nm × 7.7 nm, it contains two monomers, each of 6 nm × 5.2 nm × 3.7 nm. Each monomer has two partial actions, one part being non-covalent but tightly bound to FAD, and the other being bound to the substrate β-D-glucose. GOx at pH 3.8 has 13 lysines, which would match with the distribution of positive charges on the Taylor cone and would not affect the spinning stability. According to previous studies, this involves amide protons and H protons, with H/D exchange located on the surface of the protein, and exchange for amide II/amide I, inside the protein [86]. However, the surface proton exchange is relatively independent of the internal proton exchange. The main core reaction is still the exchange reaction inside the protein. The surface reaction connects the two monomers so that they can become an active whole. The noble metal nanoparticle DAMP was introduced as an additional energy supply, with an average size of about 2 nm [87], which is slightly larger than FAD and exactly smaller than GOx. Because of the hydrophilic layer on the surface of GOx, it exhibits a certain degree of hydrophilicity, and the hydrophilic nature of DAMP gold nanoparticles [88]. DAMP is enriched around GOx and can form good biocompatibility with GOx. Figure 9b shows a schematic diagram of DAMP for the GOx catalytic oxidation of glucose molecules, and the yellow sphere represents the ideal DAMP. DAMP relies on its own electrical conductivity and can act as an “electron transfer station” to promote the electron transmission between FAD and FADH_2_ because the efficient electron transmission between FAD and FADH_2_ can accelerate the redox reaction rate between β-D-Glucose and O_2_.

After obtaining the first-level reaction results, the surface fibers of the composite fiber membrane will dissolve to some extent. Then, the WSL in the sheath layer fibers of the core–sheath structure at the bottom will contact with water and dissolve rapidly, releasing phenol which is hydrogen-bonded to it. Phenol will be converted into benzoquinone first under the oxidation of H_2_O_2_, followed by the generation of o-benzoquinone. The color of benzoquinone is golden yellow, and the color of ortho-quinone is red. Figure 9c shows the color photos of the composite fiber membrane in glucose solutions of different concentrations after being placed for 3 h and its color matching chart. As seen in the figure, the color change deepened with increasing glucose concentration. The higher the substrate content, the faster the catalytic reaction, and the more benzoquinone production. At concentrations between 1 and 15 mmol/L, the color was close to golden yellow, indicating that mainly benzoquinone was generated within a concentration of 15 mmol/L. When the glucose concentration reached 20 mmol/L, a large amount of ortho-quinone was generated, and the color of the solution was composed of golden yellow and red, which appeared as brown on a macroscopic level. Therefore, we only performed linear fitting for concentrations between 1 and 15 mmol/L to ensure the accuracy of the data. By HSL conversion of the response color of 1–15 mmol/L, the color phase angle of each color was obtained, and its angle value was linearly fit, yielding R^2^ = 0.96707 (Figure 9c), which already includes the human blood glucose concentration range (3.9–6.1 mmol/L), meeting the needs for simple testing of human blood glucose. Today, new kinds of polymers [80,81,89], organic and inorganic nanoparticles [90,91], and novel strategies for biomedical applications [92,93,94,95,96,97] are continuously reported. These achievements can be borrowed into the design and development of electrospun nanofibers, and thus promote new kinds of sensors and biomedical devices.

## 4. Conclusions

In this study, a color-rendering sensor for glucose molecules was prepared using a sequential process of modified coaxial electrospinning and single-fluid spinning. The electrospinning processes were investigated in detail, and two kinds of fibrous films were prepared as glucose sensors. The first film F1 adopted the modified coaxial process, with the fiber core and sheath layer loading different functional substances, i.e., phenol in the core layer, and GOx in the sheath layer. The functional substances were evenly distributed in the fiber, and the GOx activity was not affected. However, the GOx in this fiber membrane could not be crosslinked and immobilized, resulting in lower efficiency of glucose degradation. The second hybrid film F2 adopted a sequential process to load phenol in the sheath of the core–sheath fiber and GOx in the single-fluid fiber, resulting in good component compatibility in the same fiber. The crosslinking of the fiber membrane enabled efficient degradation of β-D-glucose within 12 h and ultimately linear color sensing of β-D-glucose with concentrations in the 1–15 mmol/L range. The polymer-based fiber sensor with a core–sheath nanostructure used β-D-glucose as the sensing molecular model, and a fiber-efficient immobilization process was used to load and fix the GOx. By using electrospinning technology to prepare nanofiber membranes as water-soluble degradable sensors, the use of a dismountable electrospinning device makes the preparation of nanofiber membranes simple and convenient. Moreover, the resulting nanofiber-based water-soluble glucose sensors have a detection range that matches the glucose level in the human body. The hybrid film studied in this study can provide a simple template for developing sensing nanomaterials. The reaction rate can be accelerated and the reaction time shortened by adjusting the use of more effective colorimetric agents or using better catalytic peroxidase enzymes. Different processes were used to compare the impact of the structural complexity on the sensing performance of the fiber membrane. The design mode of this sequential process can apply nanotechnology directly to sensing processes, reduce excess steps and costs, and provide reference value for more sensors involving complex reaction systems.

## Figures and Tables

**Table 1:** The experimental parameters for fabricating the samples.

Film No.	Structure	Working Process	Working Fluid	Experimental Conditions ^a^
F1	Core–sheath nanofibers	Coaxial	Core fluid: 1.5 g Eudragit L 100 (EL100) was dissolved in 10 mL of anhydrous ethanol, and 2 mL of phenol was added. Sheath fluid: 0.15 g WSL was dissolved in 3 mL of deionized water; 150 mg GOx was added to 2 mL DAMP water dispersion solution. The two solutions were mixed as sheath fluid.	*V* = 10 kV *F*_c_ = 0.5 mL/h *F*_s_ = 0.2 mL/h *D* = 15 cm
F2	Hybrids of core–sheath and monolithic fibers layers	Coaxial electrospinning	Core fluid: 1.5 g EL100 in 10 mL anhydrous ethanol. Sheath fluid: 0.15 g WSL was dissolved in 3 mL anhydrous ethanol, and later 0.6 mL of phenol was added. The two solutions were mixed as sheath fluid.	*V* = 10 kV *F*_c_ = 0.5 mL/h *F*_s_ = 0.2 mL/h *D* = 15 cm
		Single-fluid blending	Working fluid: 0.8 g PVP K90 was dissolved in 10 mL of deionized water; 150 mg GOx was added to 2 mL DAMP water dispersion. The two solutions were mixed homogeneously for spinning.	*V* = 8 kV *F* = 0.5 mL/h *D* = 15 cm

^a^ The symbols of *V*, *F*, and *D* represent the applied voltage, fluid flow rate, and the nozzle of spinneret to fiber collector distance, respectively. The subscripts of c and s represent core fluid and sheath fluid, respectively.

**Table 2:** Results of the chemical composition analysis and ^31^P-NMR analysis of the WSL.

	Component (%)		Hydroxyl Group (mmol/g)			
	Lignin	Xylan	Dextran	Aliphatic Series	Condensed Phenolic Moieties	Non-Condensed Phenols
WSL	84.5 ± 0.2	5.6 ± 1.3	2.1 ± 10.6	2.8 ± 0.1	2.6 ± 0.2	1.3 ± 0.1
	84.5 ± 0.2	5.6 ± 1.3	2.1 ± 10.6	2.8 ± 0.1	2.6 ± 0.2	1.3 ± 0.1

## Data Availability

The data supporting the findings of this manuscript are available from the corresponding authors upon reasonable.
